# Familial chylomicronemia syndrome: case reports of siblings with deletions of the *GPIHBP1* gene

**DOI:** 10.1186/s12902-024-01574-9

**Published:** 2024-04-15

**Authors:** Ka Young Kim, You Joung Heo, Jung Min Ko, Young Ah Lee, Choong Ho Shin, Chang Seok Ki, Yun Jeong Lee

**Affiliations:** 1 Department of Pediatrics, Catholic Kwandong University International St. Mary’s Hospital, Incheon, Korea; 2 Department of Pediatrics, Gwangmyeong Hospital, Chung-Ang University School of Medicine, Gwangmyeong, South Korea; 3 Department of Pediatrics, Seoul National University Children’s Hospital, Seoul, Korea; 4 GC Genome, Yongin, Korea

**Keywords:** Hyperlipoproteinemias, Chylomicrons, Hypertriglyceridemia

## Abstract

**Background:**

Familial chylomicronemia syndrome (FCS) is a rare monogenic form of severe hypertriglyceridemia, caused by mutations in genes involved in triglyceride metabolism. Herein, we report the case of a Korean family with familial chylomicronemia syndrome caused by compound heterozygous deletions of *glycosylphosphatidylinositol-anchored high-density lipoprotein-binding protein 1* (*GPIHBP1*).

**Case presentation:**

A 4-year-old boy was referred for the evaluation of severe hypertriglyceridemia (3734 mg/dL) that was incidentally detected 4 months prior. His elder brother also demonstrated an elevated triglyceride level of 2133 mg/dL at the age of 9. Lipoprotein electrophoresis revealed the presence of chylomicrons, an increase in the proportion of pre-beta lipoproteins, and low serum lipoprotein lipase levels. The patient’s parents and first elder brother had stable lipid profiles. For suspected FCS, genetic testing was performed using the next-generation sequencing-based analysis of 31 lipid metabolism-associated genes, which revealed no pathogenic variants. However, copy number variant screening using sequencing depth information suggested large heterozygous deletion encompassing all the coding exons of *GPIHBP1*. A real-time quantitative polymerase chain reaction was performed to validate the deletion site. The results showed that the siblings had two heterozygous copy number variants consisting of the whole gene and an exon 4 deletion, each inherited from their parents. During the follow-up period of 17 months, the patient did not develop pancreatitis, following dietary intervention.

**Conclusion:**

These siblings’ case of familial chylomicronemia syndrome caused by rare *GPIHBP1* deletions highlight the implementation of copy number variants—beyond next-generation sequencing—as an important consideration in diagnosis. Accurate genetic diagnosis is necessary to establish the etiology of severe hypertriglyceridemia, which increases the risk of pancreatitis.

**Supplementary Information:**

The online version contains supplementary material available at 10.1186/s12902-024-01574-9.

## Background

Severe hypertriglyceridemia is defined as triglyceride levels greater than 885 mg/dL [1], which can be caused by familial chylomicronemia syndrome (FCS) or multifactorial chylomicronemia syndrome. FCS, formerly known as type 1 hyperlipoproteinemia, is a rare monogenic form of severe hypertriglyceridemia with a prevalence of 1 in 1,000,000 individuals [2]. FCS is often caused by mutations in genes involved in the chylomicron removal pathway: *LPL*, encoding the enzyme lipoprotein lipase (LPL), which is involved in 80% of FCS cases; *APOC2*, encoding apolipoprotein CII, the activator of LPL; *APOA5*, encoding apolipoprotein AV; *LMF1*, encoding lipase maturation factor 1, and *GPIHBP1*, encoding glycosylphosphatidylinositol-anchored high-density lipoprotein-binding protein 1 [3, 4]. Because patients with FCS have a high risk of life-threatening acute pancreatitis, it is essential to establish an accurate diagnosis with appropriate genetic testing [4].

GPIHBP1 is a 184-amino acid transmembrane protein that transports LPL across endothelial cells [5]. *GPIHBP1* mutation impairs transcytosis and stabilization of LPL, leading to decreased lipolysis and consequent severe hypertriglyceridemia [5]. Most *GPIHBP1* mutations that cause FCS are missense and deletion mutations have been reported in 18 cases [6–13]. In this case report, we present a Korean family with FCS resulting from deletion mutations in the *GPIHBP1* gene, aiming to expand our understanding of genetic analysis in FCS diagnosis.

## Case presentation

A 4-year-old boy was referred for evaluation of hypertriglyceridemia, which was incidentally detected four months prior (triglyceride level, 3734 mg/dL). He was born via vaginal delivery at 38 weeks’ gestation, with a birth weight of 3.7 kg and no perinatal complications; his parents were non-consanguineous. His motor and cognitive development well within normal ranges. However, he did not achieve coherent speech until he was 3 to 4 years. The patient had no personal history of pancreatitis or other medical conditions and no family history of xanthomas or early atherosclerotic cardiovascular disease. On physical examination, his height and weight were 97.4 cm (-1.8 standard deviation score [SDS]) and 15.0 kg (-1.4 SDS), respectively. There were no cases of eruptive xanthoma. The abdomen was soft and nontender without hepatomegaly. Fundus examination revealed no evidence of lipemia retinalis.

**Fig. 1:**
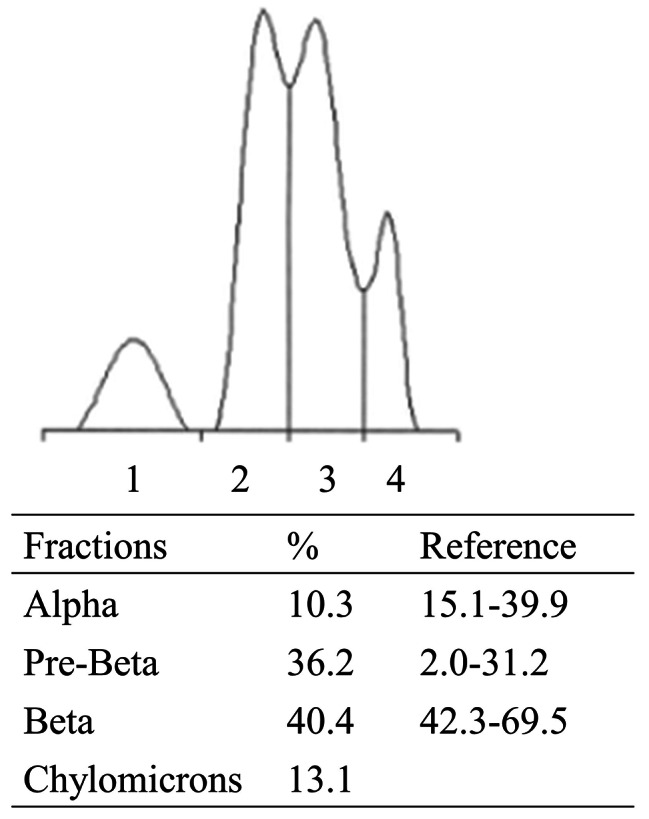
Lipoprotein electrophoresis Fraction 1, alpha lipoprotein; Fraction 2, pre-beta lipoprotein; Fraction 3, beta lipoprotein; Fraction 4, chylomicrons

Fasting biochemical tests revealed elevated total cholesterol (223 mg/dL) and triglycerides (1418 mg/dL) levels (Table 1). The high-density and low-density lipoprotein cholesterol levels were 14 and 62 mg/dL, respectively. Hepatic, renal, and thyroid function test results were within normal ranges. Lipoprotein electrophoresis revealed the presence of chylomicrons and an increased proportion of pre-beta lipoproteins (Fig. 1). LPL mass concentrations in post-heparin plasma were measured using a commercial LPL ELISA kit (LPL ELISA “DAIICHI”; Daiichi Pure Chemicals Co., Ltd., Tokyo) [14]. The post-heparin plasma LPL mass was low (57 ng/mL; reference range: 164–284 ng/mL).

**Table 1:** Laboratory findings of the sibling patients at the time of diagnosis

Variables	Values		Reference range
	Patient	Second brother	
Cholesterol (mg/dL)	223	155	< 170
Triglycerides (mg/dL)	1418	1205	< 75 (for 0–9-year-old children)
HDL cholesterol (mg/dL)	14	13	> 45
LDL cholesterol (mg/dL)	62	35	< 110
AST (IU/L)	29	22	13.0–34.0
ALT (IU/L)	12	11	5.0–46.0
Total bilirubin (mg/dL)	0.8	0.9	0.2–1.2
Albumin (g/dL)	5.1	4.6	3.3–5.2
Uric acid (mg/dL)	4.0	4.1	3.0–7.0
BUN (mg/dL)	9	17	7–17
Creatinine (mg/dL)	0.52	0.60	0.37–0.72
Calcium (mg/dL)	9.1	9.2	8.5–10.5
Phosphorus (mg/dL)	5.2	5.4	3.6–5.8
ALP (IU/L)	243	203	146–367
Free T4 (ng/dL)	1.07	1.31	0.7–1.48
T3 (ng/dL)	132.7	123.2	35–193
TSH (ng/dL)	2.5	2.4	0.35–4.94
Glucose (mg/dL)	91	81	70–110
Hemoglobin A1c (%)	5.5	-	< 5.7

HDL: high-density lipoprotein; LDL: low-density lipoprotein; AST: aspartate aminotransferase; ALT: alanine aminotransferase; BUN: blood urea nitrogen; ALP: alkaline phosphatase

The patient was suspected to have a monogenic cause of severe hypertriglyceridemia because of his young age; therefore, his brothers underwent fasting blood tests. The first brother had a normal lipid profile; however, the second, 9-year-old brother displayed severe hypertriglyceridemia (2133 mg/dL). He had no relevent medical history, including pancreatitis. The patient had no cutaneous or ocular manifestations, and his hepatic, renal, and thyroid functions were normal (Table 1). The brother’s lipoprotein electrophoresis also demonstrated the presence of chylomicrons and an increase in the proportion of pre-beta lipoproteins, and the post-heparin plasma LPL mass was low (30 ng/mL; reference range: 164–284 ng/mL). The fasting serum triglyceride levels of the father, mother, and first brother were 113, 69, and 51 mg/dL, respectively.

**Fig. 2:**
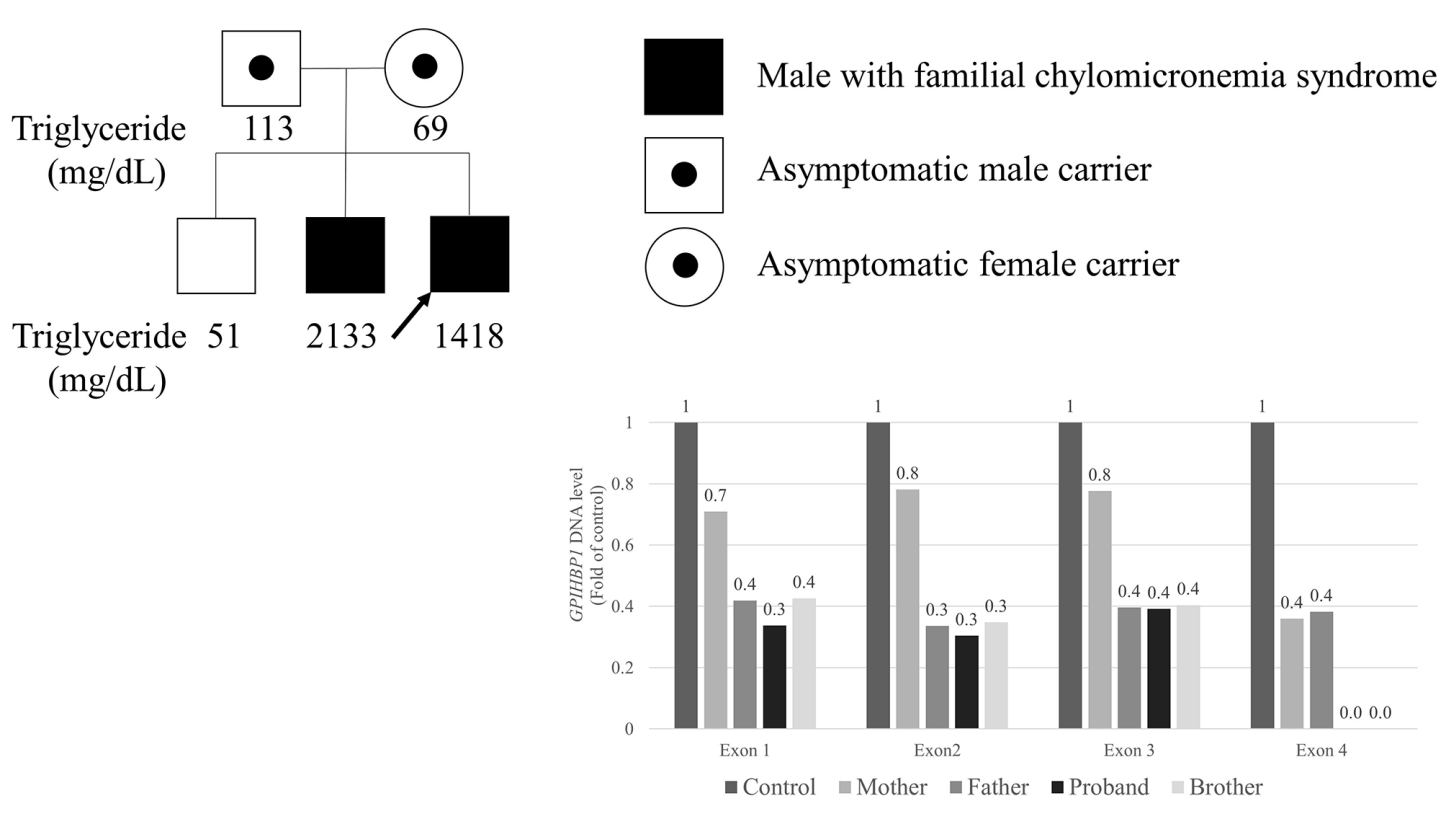
Pedigree of proband (arrow) and RT-qPCR results for the *GPIHBP1* gene This experiment was carried out twice, and the 2^-∆∆Ct^ value represents the average value from the two experiments. The 2^-∆∆Ct^ value for exons 1–3 of the proband and his brother is one-half that of the mother, and the 2^-∆∆Ct^ value for exon 4 of the sibling patients is almost zero. The 2^-∆∆Ct^ value for exons 1–4 of the father is one-half that of the control, and the 2^-∆∆Ct^ value for only exon 4 of the mother is one-half that of the control. Thus, the whole-exon 1, 2, 3, and 4 deletions in the sibling patients were derived from the father, and the exon 4 deletion was derived from the mother

On suspicion of FCS, massively parallel sequencing was performed using a molecular panel of 31 targeted genes associated with lipid metabolism disorders (Supplementary Table 1) [15]. Target enrichment was achieved via hybridization with oligonucleotide probes, and sequencing was conducted on an Illumina MiSeqDX platform (Illumina, San Diego, CA, USA). There were no pathogenic variants in previously known genes. Copy number variant (CNV) screening using next-generation sequencing (NGS) depth information suggested a heterozygous deletion encompassing all coding exons of *GPIHBP1*. As FCS is an autosomal recessive disorder, Sanger sequencing was performed to investigate variants in the counter allele; however, exon 4 amplification failed (Supplementary Fig. 1). Because an additional deletion of exon 4 on the counter allele was suspected, we performed real-time quantitative polymerase chain reaction (RT-qPCR) on both siblings and parents to confirm the deletion site around exon 4 of the *GPIHBP1* gene. Exons 1, 2, 3, and 4 were assessed using customized primers (Supplementary Table 2). The results revealed two heterozygous CNVs consisting of a whole-gene deletion and an exon 4 deletion in the patient and his second brother, which were derived from their father and mother, respectively (Fig. 2). The single-nucleotide polymorphism in the *ApoA5* and *Apo E* gene, which are known to be associated with the risk of hypertriglyceridemia, were analyzed using NGS. The *APOE* genotype was ε3ε3, and single nucleotide polymorphism rs2075291 (c.553G > T) in *APOA5* was not detected.

**Fig. 3:**
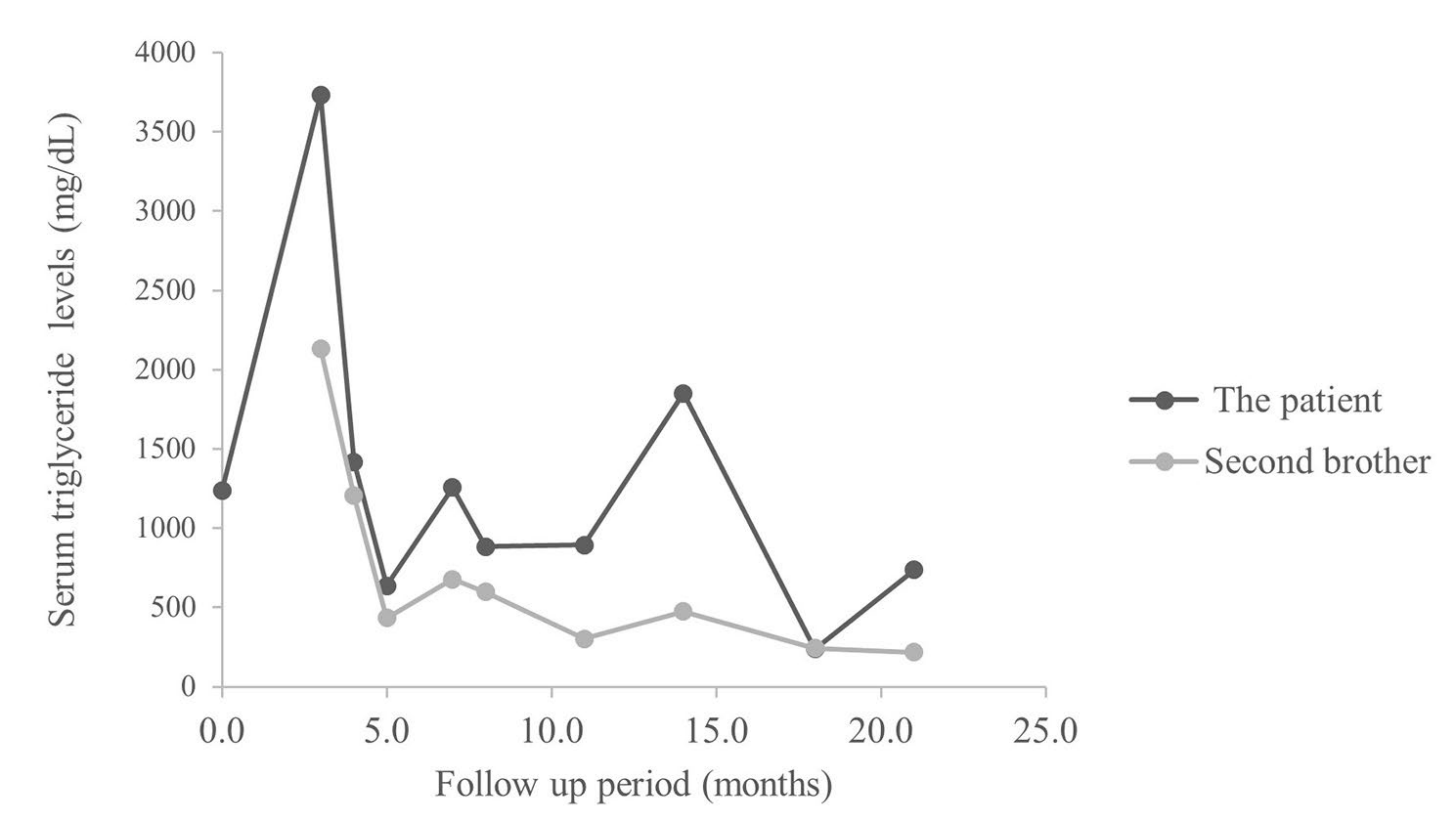
Serum triglyceride levels for the sibling patients during follow-up

The brothers initiated a low-fat and medium-chain triglyceride oil-based diet with a restriction of dietary fat intake to 15% of the total energy. Owing to the risk of acute pancreatitis associated with high plasma triglyceride levels, omega-3 acid ethyl ester (1 g) was prescribed once daily. During the 17 months of follow-up, their conditions fluctuated; however, triglyceride levels remained within the range of 200–740 mg/dL without the development of acute pancreatitis (Fig. 3). Plasmapheresis was performed because triglyceride levels decreased to < 1000 mg/dL without pancreatitis alone through dietary intervention.

## Discussion and conclusion

In this study, we report the cases of two siblings with FCS confirmed by CNV detection. The brothers carried a rare variant, a whole exon deletion and an exon 4 deletion in the *GPIHBP1* gene.

FCS is a rare autosomal recessive lipid disease with a genetic load sufficient to generate plasma triglyceride levels > 885 mg/dL due to chylomicronemia [1]. The clinical manifestations of FCS include eruptive xanthomas, lipemia retinalis, hepatosplenomegaly, and neurological symptoms [3]. The major health problem associated with FCS is recurrent acute pancreatitis, which leads to pancreatic insufficiency [16]. Thus, diagnosing FCS is important for identifying patients with severe hypertriglyceridemia and a high risk of acute pancreatitis, along with other family members who might be in the pre-symptomatic stage of the disease [4].

Our siblings had a compound heterozygous large gene deletion and an exon 4 deletion in the *GPIHBP1* gene. FCS with severe hypertriglyceridemia is caused by defects in the *LPL*, *APOC2*, *APOA5*, *LMF1*, and *GPIHBP1* [4]. Although more than 80% of FCS cases have been attributed to mutations in the *LPL* gene, mutations in *GPIHBP1* have been reported in approximately 10% of cases [6]. Human *GPIHBP1* consists of 4 exons encoding a 184-amino acid glycosylphosphatidylinositol-anchored protein that binds LPL in the subendothelial space and translocates the enzyme to the luminal surface of endothelial cells to facilitate triglyceride hydrolysis [3–5]. The C-terminus, including the LU domain, is encoded by exons 3 and 4 involved in the stabilizing the GPIHBP1-LPL complex [17]. Deletion of exon 4 produces an abnormal protein that causes a lack of circulating LPL and LPL activity in plasma [8].

Currently, there is no gold-standard diagnostic test for FCS. Genetic testing for FCS can be performed using various methods, including Sanger sequencing of a single gene, targeted exome sequencing, or whole exome sequencing. However, detecting CNVs using these methods can be challenging [18], as observed in our cases. The diagnostic yield of NGS in patients with chylomicronemia is 78% [6]. Pathogenic CNVs have been reported in *LPL*, *APOC2*, *LMF1*, and *GPIHBP1* genes [19, 20]. Deletion mutations in the *GPIHBP1* gene have been reported in approximately 5% of the cases [6], and most of their causative variants are missense mutations [9]. Therefore, if NGS results are negative for clinically suspected FCS, CNV analysis would be considered. Recently, bioinformatic tools for CNV detection from NGS data have been developed. To validate CNVs, multiplex ligation-dependent probe amplification can be employed; however, RT-qPCR can be performed as an alternative if commercial multiplex ligation-dependent probe amplification kits are unavailable [21]. Although our study has limitation that we could not accurately determine the break point, we confirmed *GPIHBP1* deletions using RT-qPCR methods.

In our cases, triglyceride levels were maintained below 1,000 mg/dL with dietary intervention, and the patients experienced no acute pancreatitis during the 2-year follow-up. In patients with *GPIHBP1* deletion mutations, the maximum triglyceride levels exhibit a wide range, from 1,100 to 37,248 mg/dL [7, 8, 10–13]. The maximum triglyceride levels of the siblings were relatively low compared to those in the other cases. Genotype-phenotype relationships in patients with FCS remain unclear. However, most cases present homozygous mutations, and most patients with acute pancreatitis harbor homozygous mutations in *GPIHBP1* [7, 8, 10–12]. The siblings in this study did not develop acute pancreatitis, possibly due to either heterozygous *GPIHBP1* mutations or early dietary interventions. Currently, the primary therapeutic strategy for FCS involves dietary fat restriction. Early intervention with a low-fat diet and medium-chain triglycerides does not fully normalize the triglyceride levels; however, it reduces the risk of pancreatitis recurrence [3, 11]. Olezarsen, antisense oligonucleotide targeting apolipoprotein C-III, might be effective in the treatment of hypertriglyceridemia without severe adverse effects [22]. A recent phase 3 randomized, double-blind, placebo-controlled study of olezarsens in adults with FCS was conducted (NCT04568434). Although the results are promising, further evidence is needed to apply this newly developed drug in children and adolescents.

In this case, we identified rare *GPIHBP1* deletions as the cause of severe hypertriglyceridemia in the siblings. CNV analysis would be considered as a second-tier test to confirm FCS if the results of sequencing analysis using an NGS panel are negative. Given the high risk of pancreatitis associated with severe hypertriglyceridemia, an accurate genetic diagnosis is important for proper management.

### Electronic supplementary material

Below is the link to the electronic supplementary material.

Supplementary Material 1

Supplementary Material 2

## Data Availability

All relevant data are included in this article.

## Abbreviations

CNV: Copy number variant
FCS: Familial chylomicronemia syndrome
LPL: lipoprotein lipase
NGS: next-generation sequencing
RT-qPCR: real-time quantitative polymerase chain reaction
