# Supplementary figures and images for: Familial chylomicronemia syndrome: case reports of siblings with deletions of the GPIHBP1 gene
Source: BMC Endocr Disord. 2024 Apr 15;24:47. doi: 10.1186/s12902-024-01574-9 (PMC11017581; doi:10.1186/s12902-024-01574-9)

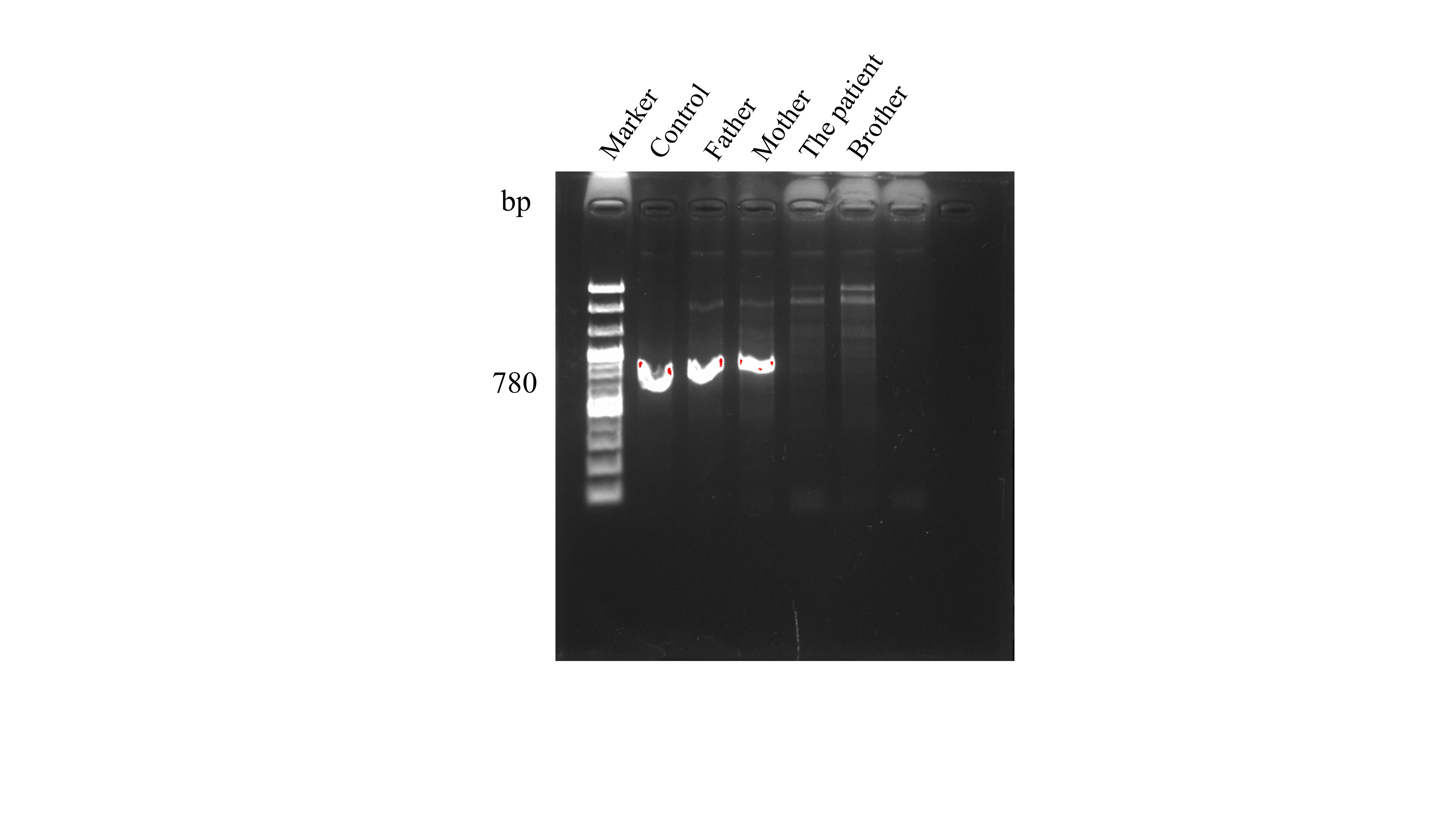

Supplement: Supplementary file 1 — Supplementary Material 1 [file 12902_2024_1574_MOESM1_ESM.jpg]
