# Supplementary material for: Familial chylomicronemia syndrome: case reports of siblings with deletions of the GPIHBP1 gene
Source: BMC Endocr Disord. 2024 Apr 15;24:47. doi: 10.1186/s12902-024-01574-9 (PMC11017581; doi:10.1186/s12902-024-01574-9)
Supplement: Supplementary file 2 — Supplementary Material 2 [file 12902_2024_1574_MOESM2_ESM.docx]

**Supplementary Table 1.** Thirty-one genes associated with lipid metabolism disorders were included in the molecular panel.

| Genes |
| --- |
| *ABCA1, ABCG5, ABCG8, ALMS1, ANGPTL3, APOA1, APOA2, APOA5, APOB, APOC2, APOC3, APOE, CETP, CREB3L3, CYP27A1, CYP7A1, GPD1, GPIHBP1, LCAT, LDLR, LDLRAP1, LIPA, LIPC, LMF1, LPL, MTTP, PCSK9, SAR1B, SCARB1, SLCO1B1, STAP1* |

**Supplementary Table 2.** Sequence of primers used for RT-qPCR.

| Primer name | Primer sequence | Size (bp) |
| --- | --- | --- |
| GPIH_1F | ACTTACCGCAGCTCCAGA | 104 |
| GPIH_1R | CAGCAAGAGGGCAAGCA |  |
| GPIH_2F | GAAGATGAGGATGAGGTGGAAG | 113 |
| GPIH_2R | GAATGCTCCAGGCAGATCA |  |
| GPIH_3F | GTGCTACACCTGCAAGTCC | 93 |
| GPIH_3R | TGGGCAATGAGGGTTGTG |  |
| GPIH_4UF | CTCCGCTAACTGTTCTCTTCTT | 88 |
| GPIH_4UR | CCCACCATCTAGGGCATTT |  |

RT-qPCR, real-time quantitative polymerase chain reaction, F: forward, R: reverse, UF: unmethylation-specific primer

RT-qPCR was performed to validate the CNVs. Primers were designed, and RT-qPCR was performed using the SYBR Green PCR Kit (Qiagen, Germany) on a 7500 Real-time PCR System (Applied Biosystems, MA). The amplification protocol consisted of an initial denaturation step at 95°C for 3 minutes, followed by 40 cycles of denaturation at 95°C for 20 seconds, annealing at 60°C for 1 minute, and extension at 72°C for 30 seconds. The quantification of the target sequence was normalized, and the relative copy number (RCN) was determined based on the comparative ΔΔCt method with normal control DNA as the calibrator. The ΔΔCt was calculated as follows: ΔΔCt = (ΔCt unknown sample - ΔCt control sample). The normalized copy number = 2ˉ^ΔΔCt^. A 0.5-fold RCN was considered a heterozygous deletion (1 copy), and an RCN of 0-fold indicated a homozygous deletion (0 copies). Each sample was assayed in triplicate to ensure the reproducibility of the results.

**Supplementary Figure 1.** PCR result of exon 3 and 4 of *GPIHBP1* analysis using the following primers: forward, 5’-CAGCACAGCTTACAGGACCA-3’, and reverse, 5’-CCAAATCCATTCTCCAAAGC-3’. 780 bp PCR product was amplified in the parents, not in the siblings.
